# Hemolytic-Uremic Syndrome in Uberlândia, MG, Brazil

**DOI:** 10.5402/2011/651749

**Published:** 2011-12-01

**Authors:** V. Bonetti, C. M. F. Mangia, J. M. F. Zuza, M. O. Barcelos, M. M. S. Fonseca, S. P. Nery, J. T. A. Carvalhaes, M. C. Andrade

**Affiliations:** ^1^Department of Pediatrics, Universidade Federal de Uberlândia, MG, Brazil; ^2^Department of Pediatrics, Universidade Federal de São Paulo, São Paulo, SP, Brazil

## Abstract

*Purpose*. To analyze the epidemiological, clinical, and laboratory characteristics of hemolytic-uremic syndrome (HUS) in Uberlandia, MG, Brazil. *Methods*. A historical cohort study was performed encompassing a ten-year period from January 1994 to January 2004 in the Department of Pediatric Nephrology at a full-service hospital; demographic factors, triggering factors, time of hospitalization, supportive therapy, and disease progression were analyzed. *Results*. Twenty-seven children aged 5 to 99 months (median age of 14 months) were studied; 70.4% were male. Of the 27 patients, 77.8% were from urban areas and 18.5% were from rural areas. Eight of the patients (29.6%) were reported to drink raw milk, and clinical diarrhea was reported in 81.5% of cases. The most common signs and symptoms were fever and vomiting (85.1%), anuria (63.0%), seizure (33.0%), cardiac involvement (11.0%), and acute pulmonary edema (7.4%). Dialysis was performed on 20 patients (74%). The mean hospital stay was 24 days (range: 13 to 36 days). While monitoring the patients, 2 died (7.4%), 3 developed chronic kidney disease (11.0%), and 21 (77.8%) developed hypertension. *Conclusion*. Our results emphasize the possibility of diagnosing HUS as a cause of renal failure in childhood in both typical (postdiarrheal) and atypical forms and suggest that an investigation of the etiological agent should be made whenever possible.

## 1. Introduction

Hemolytic-uremic syndrome (HUS), which is defined by the occurrence of microangiopathic hemolytic anemia, thrombocytopenia, and acute renal failure, was first described in children in the 1950s [1]. In Argentina, the disease is endemic and is the leading cause of acute renal failure as well as the second leading cause of chronic kidney failure [2]. In the United States, HUS is the leading cause of renal failure among children [3, 4].

Since the beginning of May 2011, an unusually high number of cases of HUS and enterohemorrhagic *E. coli* (EHEC) infections have been observed in Germany, with most cases occurring in the northern part of the country. Investigations by the German authorities indicate that the vehicle responsible for the outbreak of the verocytotoxin-producing *E. coli* (EAggEC VTEC) O104:H4 is bean and seed sprouts [5].

In 90% of cases, the etiology of HUS is associated with an infection by *E. coli* O157:H7, which is an enterohemorrhagic bacterium that produces the toxin Shiga, a virulence factor that is responsible for the intestinal and extraintestinal clinical manifestations of the disease [1].

HUS is classified as typical or atypical based on the clinical presentation. The form that is nondiarrheal (atypical) affects approximately 10% of patients, with no predilection for age; its etiology can be idiopathic or secondary. The secondary form is rare in the pediatric population and commonly occurs from pulmonary pneumococcal or meningeal infection. The idiopathic form accounts for almost all pediatric HUS cases that occur without diarrhea. Compared to the typical (post-diarrheal) form, the nondiarrheal form has a poorer prognosis and is characterized by upper respiratory tract symptoms [6].

The postdiarrheal form is considered the classic manifestation of the disease, affecting 90% of patients, who typically range in age from seven months old to six years old. The clinical presentation begins with digestive symptoms, and in the prodromal period, the main symptoms are bloody diarrhea, pain, abdominal distension, and vomiting [7].

*E. coli* is commensal with cattle, especially calves and heifers, however bovine-specific strains can cause disease in humans. Transmission of the disease is related to the ingestion of contaminated products from raw cow's milk or ruminant meat, with ground beef being the vector responsible for more than half of all reported outbreaks. Water and other food products affected by cattle feces are additional transmission vectors [8–10].

HUS is a disease that consumes a substantial amount of health care resources. Approximately 276 to 736 new cases occur annually in the United States. It is estimated that US$ 9.4 to US$ 25.2 millions are spent each year during the acute phase of illness. Every year, 24 to 63 survivors develop early-stage chronic renal failure and require transplants or constant dialysis at an additional cost of US$ 7.5 to US$ 19.1 million [3, 5].

Epidemiological data on HUS in Brazil are scarce. However, the literature has demonstrated the importance of this syndrome in terms of the endemic nature of acute renal failure in many countries, including Argentina, Canada, and Germany, as well as descriptions of epidemics such as the recent epidemic that occurred in Japan due to ingestion of contaminated vegetables [11, 12].

This study was carried out to analyze the risk factors and the clinical and presentation of HUS in a city in southwestern Brazil.

## 2. Methods

We conducted a cohort study based on institutional records of patients treated during a ten-year period (January 1, 1994 to January 31, 2004) in the pediatric nephrology department of the Hospital de Clinicas, Universidade Federal de Uberlandia, MG, Brazil.

Patients were selected for the study based on an exit diagnosis of HUS according to the discharge protocol of the Unified Health System of the Ministry of Health.

The included cases were cases of children admitted to the intensive care unit or the infirmary with acute microangiopathic hemolytic anemia, renal failure, and thrombocytopenia, which either had or had not been preceded by prodromes (diarrhea or upper respiratory tract infection).

The exploratory variables studied were age, gender, geographic origin, potential factors for the development of the disease, total hospitalization time, and the need for dialysis treatment, while the outcome variables were new comorbidity and vital status upon hospital discharge. The ethics and research committee of the institution approved the study, and participation in research was requested from the parents by means of signing an informed consent form.

### 2.1. Statistical Analysis

Demographic data are presented as percentages with either means and standard deviations or medians and 95% confidence intervals when the distribution was non-normal. The odds ratio was used to comparatively analyze survivors and non-survivors. A *P* value of less than 0.05 was considered significant.

## 3. Results

Of the patients who were treated at the pediatric nephrology clinic of the institution during the study period, 27 were diagnosed with HUS. Of these, 81.5% were from urban areas and 18.5% were from rural areas. The demographic characteristics of the patients are shown in Table 1. Eight patients (29.6%) were female and nineteen (70.4%) were male. The ages ranged from 5 to 99 months (median age 14 months), with nearly half of the patients (51.9%) between 13 and 16 months of age. The mean length of hospital stay was 25 days (6 to 70 days), and 20 patients (74.1%) underwent peritoneal dialysis (Table 1).

Analysis of triggering factors showed that diarrhea was present in 81.5% of patients, followed by upper respiratory tract infection in 18.5% and the absence of a triggering factor in 11% of cases. Ingestion of unpasteurized foods such as raw milk was reported by eight patients (29.6%) (Table 1). 

The most frequent symptoms were fever and vomiting, which were observed in 23 patients (85.1%). Nine patients (33.3%) had seizures, three (11.1%) had cardiac involvement, and two patients (7,4%) developed acute pulmonary edema (Table 2). 

During treatment, the use of hemoderivative transfusions was required in 23 cases (85.1%). Support of renal function through dialysis was undertaken in 20 cases (74.0%), and 17 patients (63.0%) had cycles of anuria during hospitalization (Table 3). 

With regard to the outcome, 2 patients (7.4%) died, 3 patients (11%) developed chronic kidney disease, and 21 (77.8%) developed hypertension (Table 1). 

## 4. Discussion 

The first reported case of HUS in Brazil was described in 1967 by Penna et al. [13]. In Brazil, reporting the occurrence of HUS is mandatory as established by Brazilian Ministry of Health Ordinance 993 on September 4, 2000. Despite this, the incidence of HUS in the country has not been established due to several factors, including underreporting and under-diagnosis of the disease in cases of forms with incomplete manifestation that are difficult to diagnose. A study reported the clinic and microbiologic features associated with 13 postdiarrheal HUS cases identified in pediatric intensive care units in the city of São Paulo, Brazil, from January 2001 to August 2005. In this paper, the combined use of microbiologic and serologic techniques provided evidence of STEC infection in 92.3% of the HUS cases studied, and the importance of O157 STEC as agents of HUS in São Paulo was highlighted [14]. 

In Argentina, there are approximately 400 new cases reported each year. From 1998 to 2005, the incidence rate increased from 8.2 to 13.9 cases per 100,000 children under the age of 5. This is due in part to mandatory reporting to the national epidemiological surveillance system since 2000, as required by the Ministry of Health [2]. 

In the city of Uberlandia, MG, we reported the presence of 27 cases of HUS, with the vast majority occurring after an episode of diarrhea. Because 9% to 30% of infections by Shiga toxin-producing *E. coli *(STEC) may develop into HUS, and 90% of cases of HUS are caused by STEC, we must always be aware of this diagnosis, especially in nursing infants who have bloody diarrhea. 

Our data on the progression of the disease, its overall treatment, dialysis, and prognosis are generally similar to those described in the literature [2, 5]. 

Hematologic manifestations of HUS after diarrhea usually disappear completely within one to two weeks of onset. The prognosis is generally favorable, with the resolution beginning after hematologic improvement. The mortality rate in the literature is less than 5%, which is slightly lower than that found in our study (7.4%) [15, 16]. 

Similar findings were also observed for permanent sequelae, such as the development of chronic kidney disease. This developed in 11% of subjects in this study, while the literature reports that 5% of patients with typical HUS develop sequelae. Serious complications affecting the central nervous system, such as seizures and stroke, are considered to be factors of unfavorable outcomes and were observed in 4 patients (14.8%) in our series. 

With regard to hypertension as a sequela of HUS, the literature reports a rate of 41.4%; however, we observed a rate of 77.8%. 

This increase in the mortality rate and the rate of sequelae in relation to other studies can be explained by the fact that our data encompassed both typical and atypical HUS, with the latter showing a less favorable progression. 

In addition to the absence of the prodrome of diarrhea, children with atypical HUS have a different mode of clinical presentation. According to a study conducted in 1993 on the clinical manifestations and outcomes of twenty children who were monitored for twenty years, there were no specific prodromes, but a significant increase in blood pressure was observed [17]. The outcome was unfavorable, given that five patients died and four developed terminal renal disease. Among the eleven patients treated with plasmapheresis, all recovered renal function at an early stage, but ten experienced subsequent clinical deterioration. 

As described above, in most patients with typical HUS, the glomerular filtration rate (GFR) returns to normal. However, it may remain reduced in 10% to 20% of patients, suggesting permanent nephron loss [18]. 

In this study, 11% of patients developed chronic kidney disease. This rate is consistent with the literature, which reports the presence of permanent sequelae in 5% to 25% of cases [19]. The prognosis of HUS has improved partly due to the early institution of supportive therapy, especially in intensive care units, as well as dialysis therapy to support renal function. 

Because typical HUS is strongly associated with certain strains of EHEC that produce Shiga toxin, such as the O157 : H7 strain, prevention of typical HUS relies in part upon measures taken to reduce the risk of *E. coli *infection. 

 Proper food preparation is essential, and an internal temperature of meat above 155 degrees F (68.3 degrees C) is necessary to eradicate contamination by EHEC. Establishing standards and educating the public in proper food preparation are important public health measures. The identification of cases, with an expanded system and more active surveillance, can lead to early detection of epidemics and the removal of possible contaminants in food that has not yet been consumed. For example, molecular epidemiology techniques helped identify an epidemic infection in Connecticut, which was linked to food from a supermarket. Supervision of hand washing by children in schools and kindergartens, as well as the exclusion of symptomatic children, can reduce the spread from person to person. Hospitalized patients should be monitored carefully, and universal precautions should be taken to prevent transmission to health workers [20, 21]. 

Although laboratory diagnosis of enterohemorrhagic *E. coli* infection can be made by stool culture, the bacteria are only present in the stools for a few days and, even if present, may not be detected by culture from stool samples. New diagnostic tools for pathogenic *E. coli* infection directly detect Shiga toxins in stool or use DNA probes to detect the toxin genes in fecal isolates. These assays are not yet clinically available. Then, the diagnosis of Stx HUS in children is generally made on clinical grounds from the characteristic clinical and laboratory findings previously described: a prodrome of diarrhea, followed by abrupt onset of the characteristic triad of microangiopathic hemolytic anemia, thrombocytopenia, and acute renal injury.

## 5. Conclusions

Our data call attention to the importance of diagnosing HUS and of researching the etiological agent whenever possible, especially in cases of diarrhea. 

Due to the severity of the disease and the possibility of underdiagnosis, there is a need to implement surveillance measures to control the incidence of STEC infections and to detect outbreaks or epidemics. Examining antibodies can greatly help in the diagnosis of STEC infection in HUS patients in whom stool cultures are negative. 

Because typical HUS is strongly associated with certain strains of EHTC that produce Shiga toxin, such as the O157:H7 strain, prevention of typical HUS relies in part on measures taken to reduce the risk of *E. coli *infection [19, 20]. 

The prognosis of HUS has improved, due in part to early institution of supportive therapy, especially in intensive care units, and to renal replacement therapy using dialysis procedures. 

## Figures and Tables

**Table 1:** Patient Demographic Characteristics.

	*N*	Frequency (%)
Number of patients	27	100
Gender		
Female	8	29.6
Male	19	70.4
Age range		
<12 months	11	40.7
12–60 months	14	51.9
61–99 months	2	7.4
Residency		
Country/unknown	5/1	18.5/3.7
Urban	21	77.8
Source of contamination		
* Unpasteurized *milk	8	29.6
Unknown	19	70.4
Time, days (median)		
Duration of hospital stay	24	13–36
Prognoses		
Chronic renal disease	3	11.1
Hypertension	21	77.7
Hospital survival	25	92.6
Mortality	2	7.4

**Table 2:** Temporal analysis related to symptoms beginning before admission.

		Age		
Prodrome^†^	Symptoms	<12 months *N* = 11 (40.7%)	12–60 months *N* = 14 (51.85%)	61–99 months
		*N*	*N*	*N*
0–3 days	Vomiting	0	1 (7%)	1 (50%)
	Fever	3 (27.2%)	2 (14.2%)	0
	Edema	1 (9%)	0 (64.5%)	0
	Oliguria	2 (18.18%)	2 (14.2%)	0

3–6 days	Vomiting	2 (25%)	4 (28.5%)	0
	Fever	2 (25%)	5 (35.7%)	0
	Edema	1 (12.5%)	2 (14.2%)	0
	Oliguria	3 (37.5%)	3 (21.4%)	0

6–8 days	Vomiting	3 (27.2%)	3 (21.4%)	0
	Fever	3 (27.2%)	2 (14.2%)	0
	Edema	0	0	0
	Oliguria	2 (18.18%)	2 (14.2%)	0

>8 days	Vomiting	2 (18.18%)	4 (28.5%)	0
	Fever	2 (18.18%)	4 (28.5%)	0
	Edema	0	0	0
	Oliguria	2 (18.18%)	3 (21.4%)	0

^†^Prodromic period distribution quartiles (Q25–Q75).

Odds ratio for vomiting between survivors and non-survivors was 0.06 (95% confidence interval (CI) 0–1.81) and for edema was 0.14 (95% CI 0.01–2.81). Odds ratio could not be calculated for the presence of oliguria and fever because there were insufficient patients to perform this calculation.

**Table 3:** Clinical variables.

	*N*	Frequency (%)
Trigger	22	81.5
Diarrhea		
Bloody	10	37
Not bloody	12	44.4
Absent/not related	4/1	14.8/3.7
Airway infection	1	3.7
Pneumonia	1	3.7
Absent/not related	2	3.7
Prodromes		
Vomiting	20	74.1
Fever	22	81.5
Edema	4	14.8
Signs and symptoms		
Oliguria/anuria	19	70.4
Renal	19	70.4
Congestive heart failure	3	11.1
Convulsion	9	33.3
Acute edema	2	7.4
Hypertension	22	81.5
Dialysis/transfusion	20/23	74.1/85.2

^†^Interquartile range.
